# A meta-analysis of chemokines in alopecia areata: recruiting immune cells toward the hair follicle

**DOI:** 10.3389/fimmu.2025.1648868

**Published:** 2025-09-03

**Authors:** Elise Van Caelenberg, Arno Belpaire, Nanja van Geel, Reinhart Speeckaert

**Affiliations:** ^1^ Department of Head and Skin, Faculty of Medicine and Health Sciences, Ghent University, Ghent, Belgium; ^2^ Department of Dermatology, Ghent University Hospital, Ghent, Belgium

**Keywords:** alopecia areata (AA), chemokine, pathogenesis, CXCL, biomarker, scoping review, meta-analysis, Th1 & Th2

## Abstract

A deeper understanding of the immune-based pathogenesis of alopecia areata is essential for the development of novel targeted therapies. Compared to cytokines, chemokines exhibit substantially higher serum concentrations, offering a more robust approach for large-scale immune profiling. However, the complexity of chemokine interactions presents challenges in defining their precise roles in AA. To explore these dynamics, we conducted a scoping review and meta-analysis of 46 original research articles examining chemokine expression in skin and blood samples from AA patients; meta-analysis was performed when three or more studies assessed the same chemokine in comparable groups. Th1-associated chemokines—including CXCL9, CXCL10, CCL5, and CXCL11—were consistently elevated in AA, reflecting the known IFN-γ–driven response. A distinct Th2 chemokine signature was also observed, with increased levels of CCL13, CCL17, CCL22, and CX3CL1. Additionally, elevated levels of CCL2, CCL3, CCL4 (monocyte/dendritic cell recruitment), and CCL11, CCL24, and CCL26 (eosinophil recruitment) suggest the involvement of immune pathways beyond classical T helper subsets. Meta-analysis confirmed significantly elevated serum levels of CXCL9 (p = 0.003), CXCL10 (p = 0.004), CXCL8 (p < 0.001), and CCL17 (p < 0.001). These findings reveal a complex chemokine profile in AA, dominated by Th1 activity but also implicating Th2 and other immune pathways, highlighting the potential benefit of broader immunomodulatory strategies to address the multifaceted immune dysregulation underlying the disease.

## Introduction

Alopecia areata (AA) is the most common autoimmune cause of non-scarring hair loss, with a lifetime incidence of approximately 2%. Hair loss can range from small patches (patchy type) to complete loss of scalp (alopecia totalis) or body hair (alopecia universalis). Despite being non-scarring, AA often follows a chronic course, and spontaneous recovery in alopecia totalis occurs in only 16% of cases without treatment. Moreover, severe AA often requires long-term treatment, with frequent relapses during dose tapering.

Although the exact mechanisms are not fully understood, growing evidence points to a central role for immune-mediated pathways in its pathogenesis. Specifically, dysregulated cytotoxic T cells and natural killer (NK) cells appear to mediate an autoimmune attack on hair follicles, driven by an IFN-γ–dominant response. This leads to pronounced perifollicular inflammation, which disrupts the hair growth cycle and impairs follicle function without causing permanent damage or scarring (1). Despite this Th1-dominant profile, AA shows a notable association with atopy, particularly atopic dermatitis, which is the most common comorbidity. Interestingly, treatment responses also differ by atopic status: only patients with elevated IgE levels tend to respond well to dupilumab, with a SALT75 response rate of 50% after 72 weeks, compared to just 8% in those with low IgE levels. This highlights the complex and heterogeneous inflammatory signaling involved in AA pathogenesis (2, 3).

A key initiating factor in the autoimmune attack on hair follicles in AA is the collapse of hair follicle immune privilege (IP)**—**a local protective mechanism that normally shields follicles from immune recognition (4). In AA, this barrier breaks down, allowing immune cells to infiltrate and target the follicle. This infiltration is largely orchestrated by chemokines, small signaling proteins that guide immune cells to specific tissues, including the skin (5). With approximately 50 chemokines and 18 receptors identified, the chemokine system exhibits overlapping functions, making it a highly redundant and complex signaling network (6).

In AA, chemokines such as CXCL9, CXCL10, and CXCL11 are upregulated in lesional follicles under the influence of IFN-γ via the JAK–STAT pathway. These chemokines act through their common receptor CXCR3 to orchestrate directed recruitment of the cytotoxic Th1-type lymphocytes into the perifollicular region, amplifying local cytotoxic inflammation (2, 7). In murine AA models, pharmacological blockade of CXCR3 prevents disease onset, underscoring the pathogenic role of this axis (8).

Chemokines therefore form pivotal bridges between the inflammatory cytokine milieu (e.g. IFN-γ) and recruitment of effector and antigen-presenting cells to the hair follicle. They disrupt local immune privilege and initiate a feed-forward cycle of cytotoxic T-cell infiltration and follicular damage—providing mechanistic insight into how immune responses translate into clinical hair loss in AA.

Due to their rapid responsiveness to immune changes and higher circulating levels compared to cytokines, chemokines have emerged as valuable biomarkers in autoimmune diseases like AA. Their short half-life enables real-time monitoring of immune activity; however, this also makes them sensitive to transient fluctuations, such as those caused by infections, which can obscure the underlying chronic inflammation in autoimmune conditions (9, 10).

The large diversity of chemokines and their complex interactions pose significant challenges in understanding their precise role in AA. This scoping review aims to systematically map the current evidence regarding the involvement of chemokines in AA. By exploring their role in the disease pathogenesis, their potential as biomarkers, and the therapeutic implications, this review seeks to provide a comprehensive overview of existing research and identify gaps that warrant further investigation.

## Materials and methods

A systematic search was conducted in PubMed and Embase to identify all articles investigating chemokines in AA, with the aim of comparing levels between patients and healthy controls, as well as between lesional and non-lesional skin. All articles from inception to 15 November 2024 were screened for eligibility. The search strategy included the keywords “alopecia areata AND (chemokine OR chemokines)” in all fields. One article, published in February 2025, was added afterwards as it replaced an abstract that had been included during the initial search in November 2024, once the full text became available (11). Only human studies investigating chemokine levels in the blood and/or skin of AA patients were included, while animal models were excluded. Full-text articles, short manuscripts, letters and abstracts were all considered, and all languages were allowed. Duplicates were removed based on similar content and authorship. The following comparisons were extracted: comparison between AA and healthy controls and between lesional and non-lesional skin. Comparisons of non-lesional skin versus healthy skin were not taken into account. *In vitro* models were also excluded given their uncertain capacity to reflect the *in vivo* chemokine profile. The extracted data included the detected chemokine levels with their statistical significance, the number of patients, the method to measure chemokines (e.g., ELISA, RNA analysis,.). Meta-analysis was done in case at least 3 articles performed the same analysis at the protein or RNA level in comparable patient groups. The meta-analysis was carried out with Review Manager 5.4.1 (The Cochrane Collaboration, 2020) using an inverse variant random effects model with the standardized mean difference as an effect measure. The standardized mean difference was chosen instead of the mean difference due to substantial variability in baseline chemokine values among healthy controls, reflecting differences in laboratory kits and evaluation techniques. The mean chemokine concentrations, standard deviation, and number of patients were extracted from each publication. In case only the sample size, range, median, and/or interquartile range were mentioned, the mean was calculated by the formula of Luo et al., 2018 and the standard deviation was calculated based on Wan et al., 2014 (4, 5). If studies displayed the results in graphs without providing the exact values, data were extracted with GIMP 2.10.30 (GNU image manipulation program) using the methodology published by Van der Mierden et al., 2020. If mean values and standard deviations were not reported and no graphs were available, the data were calculated from the median and range using the formula by Hozo et al. (2005) (12).

## Results

**Figure 1:**
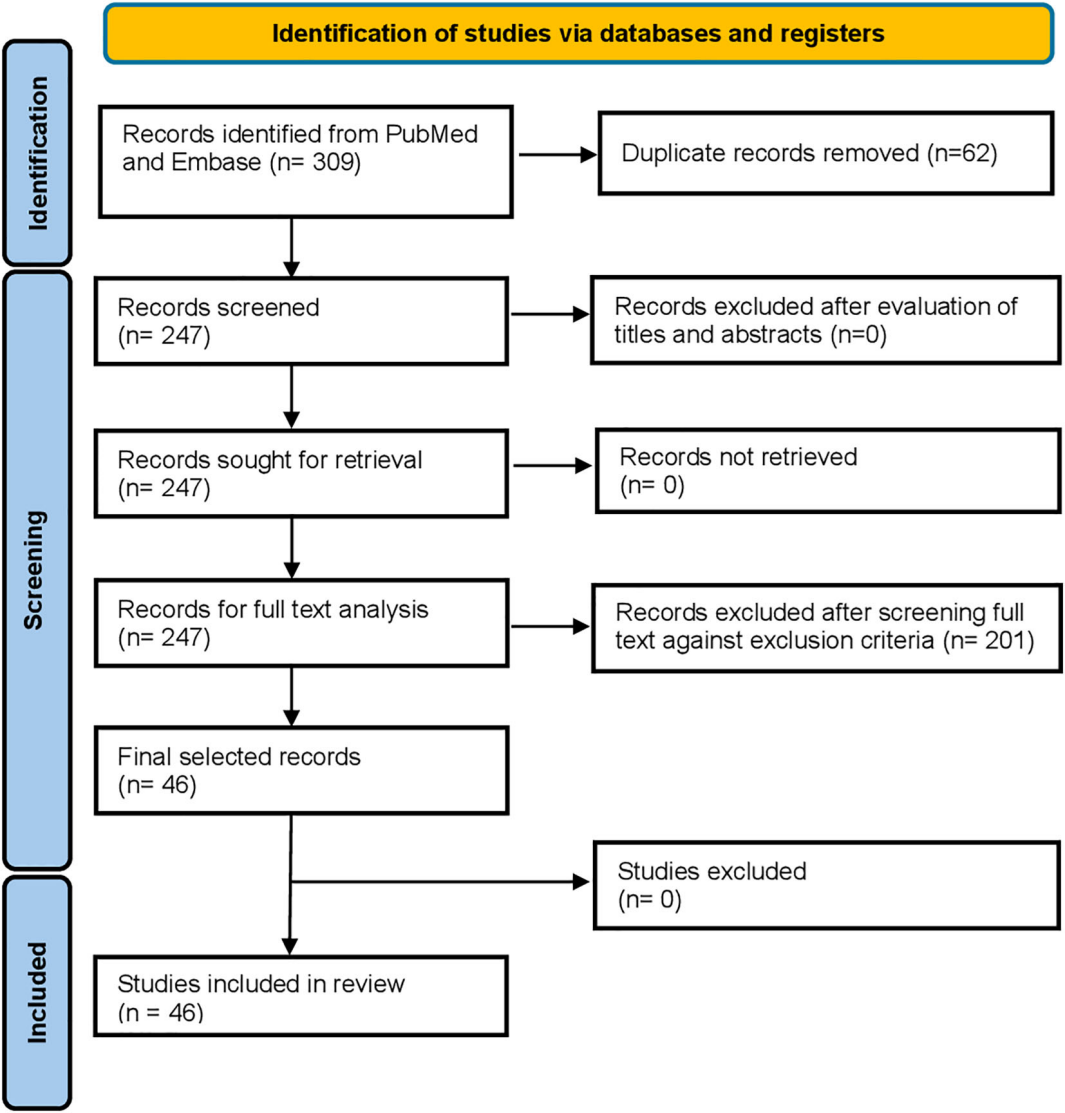
PRISMA flow diagram of the study selection process.

A total of 309 records were identified through database searching, of which 46 met the inclusion criteria. The selection process is illustrated in **Figure 1**.

Among the 46 included studies, 24 reported chemokine levels in blood (protein, RNA, or DNA), 21 in skin (protein or RNA), and one study reported on both (13).

Most results on chemokines in the circulation were found for CCL17 (n=7), CXCL9 (n=7), CXCL8 (n=6), CXCL10 (n=6), CCL5 (n=6), CCL4 (n=6), CCL7 (n=5), CXCL1 (n=5), and CCL2 (n=5) followed by CCL13 (n=4), CCL3 (n=4), CCL11 (n=3), and CCL20 (n=3). The remaining chemokines were only reported by one or two sources.

Similar results were found for chemokine reports in the skin: most results were found for CXCL10 (n=16), CXCL9 (n=12), CCL5 (n=10), CCL13 (n=8), CCL18 (n=8), CXCL11 (n=6), CCL2 (n=5), CXCL8 (n=4), CCL22 (n=4), CCL17 (n=4), CCL26 (n=4) CXCL1 (n=3), CXCL13 (n=3), CCL19 (n=3), CX3CL1 (n=3),CCL8 (n=3), and CCL20 (n=3).

**Figure 2:**
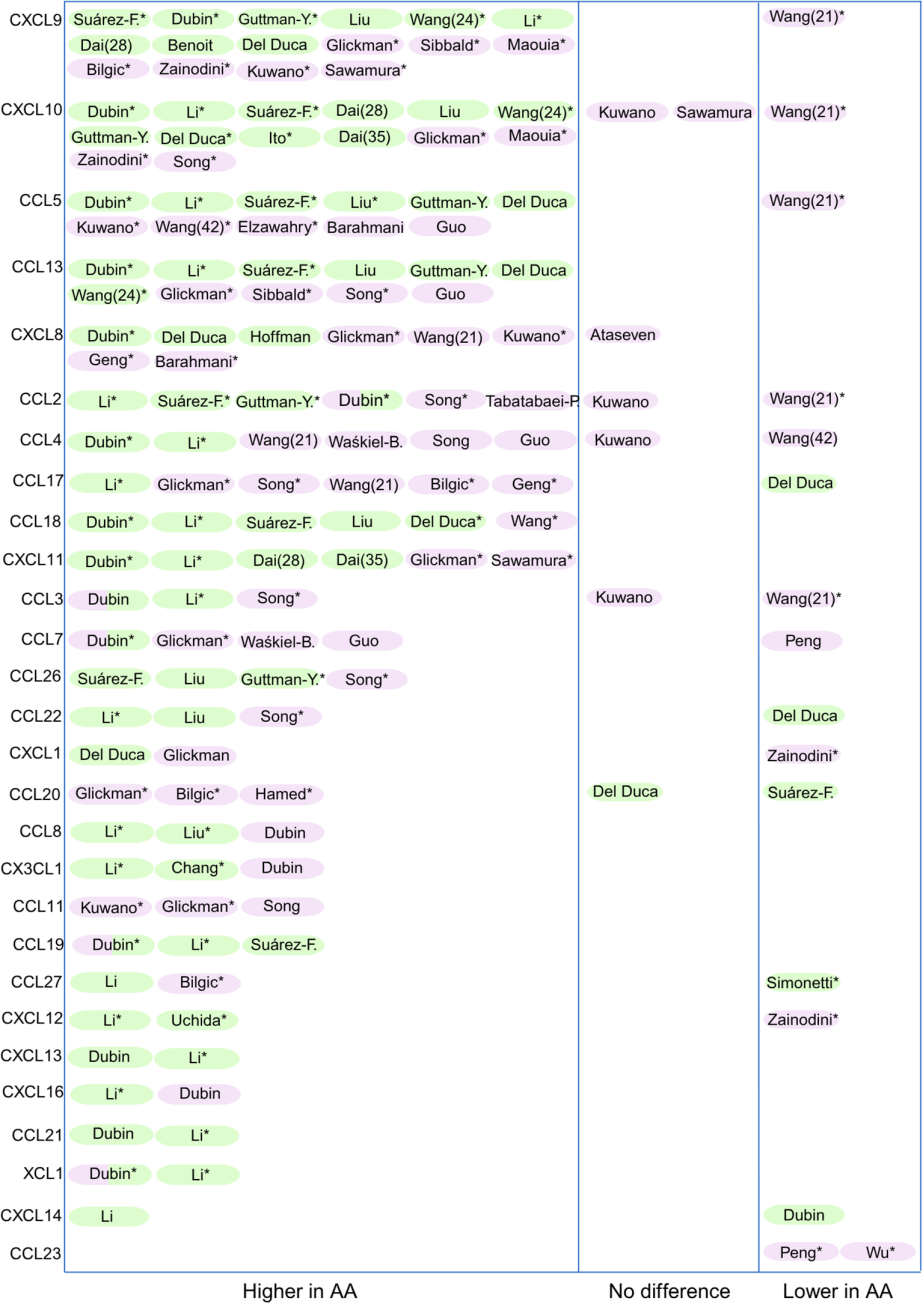
Overview of studies reporting chemokine expression in AA in skin (green) and blood (purple). Only chemokines that were assessed in more than one study comparing AA patients to healthy controls are shown (n = 30). The remaining 8 chemokines (CCL16, CXCL17, XCL2, CCL25, CXCL3, CCL28, CXCL5, and CXCL2) were excluded due to limited reporting. *= significance was reached (ranging from p<0.1 to p<0.0001). The authors who contributed to more than one article (Wang and Dai) are identified by their corresponding reference numbers in parentheses.

35 out of the 46 studies directly compared chemokine levels in AA patients to healthy controls. **Figure 2** provides an overview of these articles. Chemokines are listed from top to bottom, which reflects the strength of evidence: chemokines with the most reports appear at the top, and those with limited data appear lower down. The remaining 11 articles focused on comparisons between lesional and non-lesional skin, investigated associations with disease severity (SALT score), or assessed changes in chemokine levels following treatment.

### Chemokines primarily related to the adaptive immune system

#### Th1-related chemokines

##### CXCL9

**Figure 3:**
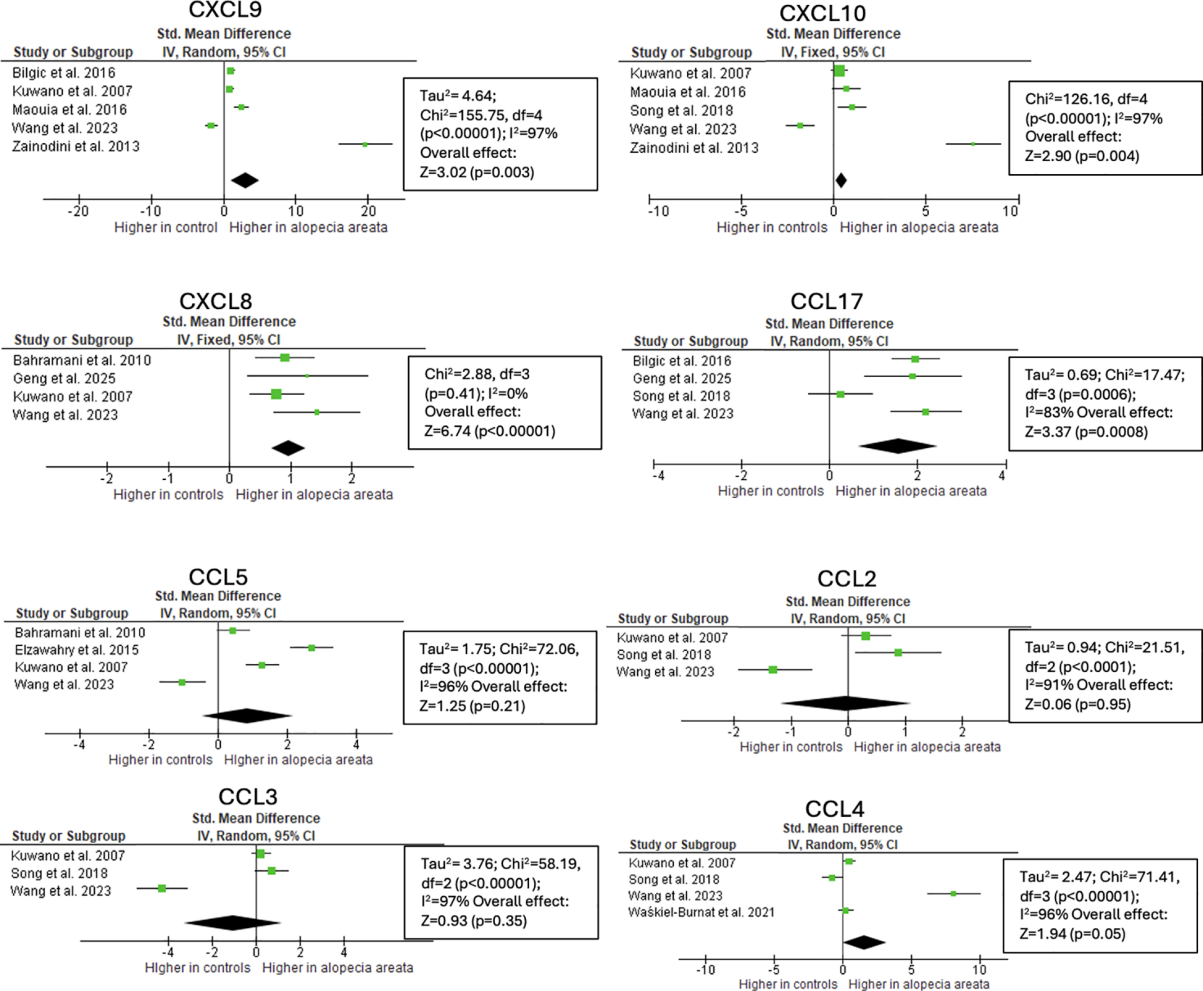
Meta-analysis of circulating chemokines of AA patients versus controls. CXCL9 (p=0.003), CXCL10 (p=0.004), CXCL8 (p<0.001), CCL17 (p<0.001) were significantly higher in AA patients. CCL, chemokine (C-C motif) ligand; CXCL, chemokine (C-X-C motif) ligand.

CXCL9 has been extensively studied in AA (501 patients vs 358 healthy controls), with 7/8 studies reporting increased CXCL9 concentration in the circulation of AA patients compared to controls (14–21). This was confirmed by meta-analysis, showing a standardized mean difference (SMD) of 3.08 (95% CI: 1.08–5.08; p = 0.003) (**Figure 3**). In the skin, 9 out of 9 studies reported upregulation of CXCL9 in AA compared to healthy controls (2.63–36.9-fold increase). Used techniques were microarray (n = 4), RT-PCR (n = 2), RNA sequencing (n = 2), and mRNA *in situ* hybridization (n=1) (13, 22–28).

Six studies compared lesional to non-lesional skin (125 lesional vs 96 non-lesional), with overall higher CXCL9 RNA expression in lesional skin, although the difference was smaller than compared to healthy controls and significance was not always reached (22, 23, 25, 27, 29, 30).

Although baseline lesional CXCL9 levels did not correlate with the SALT score, a strong positive correlation (r = 0.91, p = 0.03) was observed between changes in lesional CXCL9 levels and changes in the SALT score following effective dupilumab treatment. This association was found in a cohort of both atopic and non-atopic patients (29, 31). Similarly, although several studies reported no correlation between serum CXCL9 levels and AA severity at a single time point, one study observed significantly elevated levels during disease exacerbation and decreased levels during remission (p<0.01), suggesting a dynamic association with disease activity (16, 17, 19).

##### CXCL10

CXCL10 levels in serum were reported by 6 studies (316 patients vs 190 healthy controls). The used techniques were ELISA, multiplex assays, flow cytometry, O-link technology, and serum droplet PCR. 3/6 studies reported a significant increase in AA compared to healthy controls (80 patients vs 81 controls) (14, 16, 18). 2 articles reported no difference and 1 article reported lower values in AA after Covid vaccination compared to patients without AA after vaccination (25 patients vs 17 controls) (19–21). Overall, the meta-analysis revealed that serum CXCL10 concentrations were significantly higher in patients with AA compared to healthy controls, with a SMD of 0.44 (95% CI: 0.14–0.74; p = 0.004) (**Figure 3**). Individual study results varied notably, ranging from a large negative effect size in AA after Covid vaccination to an extremely large positive effect size.

CXCL10 expression in the skin was reported by 11 studies, all showing consistently higher CXCL10 in lesional AA skin compared to healthy control skin (205 lesional vs 123 controls). Techniques included microarray, RT-PCR, RNA sequencing, and immunofluorescent staining. Six of these studies reported fold changes ranging from 3.7 to 34.00 (13, 22–25, 32).

6 of these 11 studies also compared lesional to non-lesional skin (79 lesional vs 69 non-lesional). Increased CXCL10 RNA was found in lesional skin although the difference was less compared to healthy controls and significance was not always reached (22, 23, 25, 27, 30, 33).

Although baseline lesional CXCL10 levels did not correlate with the SALT score, a strong positive correlation (r = 0.84, p = 0.08) was observed between changes in lesional CXCL10 levels and changes in the SALT score following effective dupilumab treatment (29, 31). 2 articles on serum showed no correlation between serum CXCL10 and SALT (16, 31).

##### CXCL11

Compared to CXCL9 and CXCL10, the expression of CXCL11 seems less pronounced in skin diseases (34). Unlike the pro-inflammatory CXCL10, CXCL11 promotes the development of IL-10–producing regulatory T cells, which help control autoimmunity (6). In AA, CXCL11 has little data including 6 studies with in total 156 patients and 111 controls. Only 1 study focused on the serum protein level with a 1.5 fold increase in AA compared to healthy controls (p<0.05) (14). Cell-free DNA of CXCL11 was 18% higher in AA compared to healthy controls and 4 studies showed higher CXCL11 in AA skin compared to healthy control skin (13, 20, 27, 28, 35). No studies reported correlations with disease severity or SALT score.

##### CXCL16

This IFN-γ– inducible chemokine was not increased in serum in 1 study, but was significantly increased in AA tissue (13, 27).

##### CCL5 (RANTES)

CCL5 is a chemokine induced by IFN-γ and IL-2. Although it has pleiotropic effects and is involved in various immune pathways, it is commonly associated with Th1-type responses (36–38). 12 studies detected increased lesional or serum levels, 7 of them significantly (13, 19, 22, 23, 25–27, 39–43). Despite some studies showing elevated circulating CCL5 levels in AA, the overall effect was not statistically significant, mainly due to 1 study that showed significant lower concentrations in AA induced after COVID vaccination (**Figure 3**).

5 studies investigated the association between CCL5 expression and disease severity. In skin samples, results were mixed: while baseline lesional CCL5 expression did not correlate with the SALT score, a strong positive correlation (r = 0.81, p = 0.1) was observed between changes in lesional CCL5 levels and changes in the SALT score following effective dupilumab treatment (29, 31). In blood, CCL5 was not correlated with lesion extent at a single time point, but levels were significantly elevated with increasing disease activity (19, 39, 43).

#### Th2 related chemokines

##### CCL13

CCL13 induces chemotaxis of lymphocytes, eosinophils, basophils and monocytes. It has been implicated in allergic responses such as asthma (44). 5/5 studies on serum found higher CCL13 levels in AA compared to healthy controls (85 patients vs 63 controls), which was confirmed on skin by 7/7 studies (174 patients vs 125 controls) (13–15, 22–27, 31, 39). 3 studies investigated the correlation between CCL13 levels and SALT scores. In skin samples, 1 study found a non-significant weak to moderate correlation (r=0.34, p=0.069), while another observed a stronger correlation with changes in SALT scores after dupilumab treatment (29, 31). In serum, one study reported a correlation with AA severity, whereas another did not (31, 39).

##### CCL17

CCL17 binds to the CCR4 receptor expressed by Th2 cells and regulatory T cells, thereby attracting these cells to the hair follicle (45). CCL17 protein levels in serum were assessed in 6 studies (11, 14, 17, 21, 31, 46). Although with varying levels of significance, all studies reported increased CCL17 levels in patients with AA compared to controls. This trend was further supported by meta-analysis, which revealed significantly higher serum CCL17 concentrations in AA patients, with a SMD of 1.56 (95% CI: 0.65–2.47; p = 0.0008) (**Figure 3**). Additionally, 1 article showed significantly higher levels in 16 alopecia totalis or universalis patients compared to 40 patients with mild patchy AA (47).

Only two studies compared CCL17 levels in AA skin with healthy skin: one found significantly elevated levels, while the other observed a non-significant decrease (22, 27). 3 studies examined CCL17 levels during and after therapy: 2 reported a decrease in skin and serum CCL17 levels following effective dupilumab treatment, while one study found no change in CCL17 skin expression after successful treatment with intralesional steroids (29, 30, 46).

Two studies investigated the association between scalp CCL17 levels and SALT scores. One found no correlation, while the other reported a strong correlation (r ≥ |0.8|, p ≤ 0.1) between changes in lesional CCL17 levels and SALT scores following effective dupilumab treatment (29, 31). In serum, one study found no correlation, while another reported a significant correlation with an odds ratio of 1.095 (95% CI: 1.013–1.183, p = 0.021) (17, 31).

##### CCL22

CCL22 is closely related to CCL17 and known as macrophage-derived chemokine (MDC). It acts on CCR4-expressing cells including Th2 and Tregs (45). CCL22 was higher compared to healthy controls in 2 mRNA studies and in 1 study on serum (26, 27, 31). Only one study examined the association between CCL22 levels and SALT scores in both skin and serum. While no association was found in serum, qRT-PCR revealed a strong correlation (r ≥ |0.8|, p ≤ 0.1) between changes in lesional CCL22 levels and SALT scores following effective dupilumab treatment (29).

##### CX3CL1

A third related chemokine, CX3CL1, affects monocytes, NK cells, and T cells (48). Its levels were found to be elevated in the skin in two studies and in serum in one study (8, 13, 27). Furthermore, CX3CL1 was found to be higher in lesional skin compared to non-lesional skin in AA patients (33).

#### Th17 related chemokines

##### CCL20

The lymphocyte and neutrophil attracting CCL20 was found to be 1.5 to 2 times higher in serum of AA patients compared to healthy controls, according to 3 studies involving 155 patients and 116 healthy controls, all of which reached statistical significance (14, 17, 49). In contrast, data on lesional CCL20 expression have been more inconsistent. One study reported similar CCL20 levels in AA skin and control skin, although the sample size was limited (8 patients vs 8 controls), while another study found non-significant lower concentrations in AA patients (22 patients vs 3 controls). Analysis of CCL20 levels in relation to SALT scores showed no correlation with disease severity in either skin or serum samples (17, 29).

#### B lymphocyte related chemokines

##### CXCL13

Lesional mRNA levels of the B lymphocyte recruiter CXCL13 were elevated in two studies, with one study also reporting significantly higher expression in lesional compared to non-lesional skin (13, 27).

#### Other lymphocyte-attracting and lymphoid tissue-associated chemokines

##### CCL21 and CCL25

CCL21 recruits T cells into secondary lymphoid organs and was studied on tissue in 2 studies, both showing increased values in AA (13, 27). CCL25, involved in lymphopoiesis and lymphoid organ development was studied in 1 report and showed non-significant increased values in AA (14).

##### CCL19

CCL19, or macrophage inflammatory protein-3-beta (MIP-3β), which recruits dendritic cells and B cells, was reported to be elevated in alopecic skin in three studies and in serum in one study (1.38-fold) (13, 25, 27).

##### CCL27

CCL27, a T memory cell recruiter, was reported to be twice as high in serum levels of AA patients compared to healthy controls in one study (17). In the skin, one study found significantly lower immunohistochemical expression of CCL27 in AA skin, while another study observed no difference overall, except for a higher expression in lesional compared to non-lesional skin (27, 50). Only one study examined the association with SALT scores, reporting a significant positive correlation in serum samples based on logistic regression (odds ratio: 1.011; 95% CI: 1.002–1.020; p = 0.017) (17).

### Chemokines primarily related to the innate immune system

#### Eosinophils

##### CCL11 (eotaxin-1)

3 studies showed an increase of the eosinophil recruiting chemokine CCL11 in blood of AA patients compared to healthy controls (150 patients vs 74 controls) (14, 19, 31). No data on skin was reported. Two studies examined the correlation between serum CCL11 levels and disease severity. One reported a moderate positive correlation with the SALT score (r = 0.45, p = 0.013), while the other found no significant correlation (19, 31).

##### CCL24 (eotaxin-2) and CCL26 (eotaxin-3)

Only 2 studies on CCL24 were reported. One showed a slight (1.3-fold) non-significant increase in serum protein concentration in AA compared to healthy controls and the other found decreased scalp mRNA levels in AA after dupilumab treatment (-2.1-fold, no significance) (14, 29).

Regarding CCL26, one study reported significantly higher serum levels in AA patients compared to healthy controls (30 patients vs. 10 controls (31). Additionally, CCL26 levels were elevated in lesional skin compared to healthy skin (1.6–7.3-fold, p < 0.05) and to non-lesional skin (4.54–7.83-fold, p < 0.01) (23, 25, 26). Following treatment with ustekinumab or dupilumab, CCL26 levels significantly decreased by -2.17-fold and -3.93-fold, respectively (23, 29). In skin, changes in lesional CCL26 levels strongly correlated with changes in SALT scores after effective dupilumab treatment (r ≥ |0.8|, p ≤ 0.1), while in serum, no association with SALT scores was observed (29, 31).

#### Neutrophils

##### CXCL8 (IL-8)

The potent neutrophil-recruiting chemokine, CXCL8, was found to be twice as high in blood samples from AA patients compared to healthy controls in five out of six studies, while 1 article reported no difference (11, 14, 19, 21, 40, 51). These findings were confirmed by meta-analysis, showing significantly elevated serum CXCL8 levels in AA patients, with a SMD of 0.97 (95% CI: 0.69–1.25; p < 0.00001) (**Figure 3**). This trend was further supported by the 3-to-5-fold increased levels in lesional compared to healthy skin and the significant associations between serum CXCL8 levels and the SALT score (11, 13, 19, 22, 52).

##### CXCL1

Mixed results were reported for the neutrophil chemoattractant CXCL1. In serum, one study observed a slight non-significant increase (1.13-fold) in AA patients compared to healthy controls, while another reported significantly lower levels in AA (14, 18). In the skin, lesional CXCL1 levels were non-significantly elevated in AA compared to controls (1.4-fold) (22, 33). One study investigated the correlation between CXCL1 levels in the skin and the SALT score but found no significant association (29).

##### Other chemokines

Another neutrophil attractant, CXCL2, was significantly increased in AA skin and lowered significantly after dupilumab treatment (27, 29). CXCL5, also termed epithelial-derived neutrophil-activating peptide 78 (ENA-78) was 1.19-fold increased in AA, though not significantly (14).

#### Monocytes, macrophages, dendritic cells

##### CCL2

Mixed results were reported for CCL2. Three studies found a 1.2-fold increase in serum protein levels in AA patients while another study observed no difference, and one study reported lower levels in patients who developed AA following Covid vaccination (13, 19, 24, 31, 53). The meta-analysis showed no significant difference in serum CCL2 concentrations between patients with AA and healthy controls (**Figure 3**). In contrast, 5 studies consistently showed elevated CCL2 mRNA in AA skin, confirming its contribution to the inflammatory signaling (13, 23, 25, 27, 41). Only 1 study examined the correlation between CCL2 levels and SALT scores, reporting a weak, non-significant correlation (r = 0.23, p = 0.22) in serum samples (31).

##### CCL3

Similar results were observed for CCL3, a chemokine that attracts macrophages, monocytes, and neutrophils (54). Two studies reported an increase in serum protein levels in AA, while another study observed no difference, and one study reported lower levels in patients who developed AA following Covid vaccination (13, 19, 21, 27, 31). The meta-analysis did not reveal a statistically significant difference in serum concentrations between patients with AA and healthy controls (**Figure 3**). One study examined the correlation between serum CCL3 levels and SALT scores, reporting a weak, non-significant correlation (r = 0.22, p = 0.24) (31).

##### CCL4

Serum levels of Macrophage inflammatory protein-1β (MIP-1β) (CCL4) were elevated in AA patients across four studies, although not significantly (21, 31, 39, 55). This trend was supported by the meta-analysis, which showed an SMD of 1.61 (95% CI: -0.01 to 3.23; p = 0.05), approaching statistical significance (**Figure 3**). Statistical significance was achieved in the skin, with a fold increase of 4.5 (13, 27). Both studies that assessed the relationship between serum CCL4 levels and SALT scores found no correlation (31, 55). The migration of dendritic cells is also regulated by CXCL17, which was significantly higher in AA skin (2.7-fold) (13).

##### Other chemokines

CCL16 binds to multiple receptors on monocytes, macrophages and Th2 cells and was studied by one source showing a non-significant increase in AA (1.2-fold) (13). Mixed results were found for CXCL14 which is produced by fibroblasts and interacts with monocytes and dendritic cells (56). One study reported increased lesional mRNA in AA while another study did not detect any difference (13, 27). CXCL3 or macrophage inflammatory protein-2-beta (MIP-2β) was not significantly elevated in AA scalp tissue (1.09-fold), but was significantly elevated when comparing lesional to non-lesional biopsies (1.70-fold, p<0.05) (25).

### Pleiotropic chemokines

#### CCL7

CCL7 recruits many inflammatory cells (lymphocytes, dendritic cells, eosinophils, neutrophils, NK cells) (55). Four studies reported a slight increase in its levels in the circulation of AA patients, which was further confirmed in scalp tissue by one study (13, 14, 39, 55). Two studies examined the correlation between serum CCL7 levels and SALT scores, both reporting a positive correlation, with one providing specific data (r = 0.281, p = 0.03) (39, 55).

#### CCL8

CCL8 is a pleiotropic chemokine activating many different immune cells (57). Although only a slight increase in serum levels was observed (1.26-fold), 2 studies reported significantly higher scalp levels (13, 26, 27).

#### CCL18

In contrast to the other chemokines that are upregulated by IFN-γ, CCL18 is downregulated by IFN-γ and recruits a variety of immune cells (58). 6 studies pointed all to increased expression both in the skin and the circulation (13, 21, 22, 25–27). Two studies examined the association between CCL18 and SALT scores. One reported a strong, significant correlation between changes in lesional CCL18 levels and SALT scores following effective dupilumab treatment (r ≥ 0.8, p < 0.05), while the other found no significant correlation (29, 31).

#### CCL23

CCL23, a chemoattractant for lymphocytes, monocytes and neutrophils was inversely correlated with AA according to two Mendelian Randomization IVW analyses (59–61).

#### CXCL12

CXCL12 is primarily a homeostatic chemokine that regulates the steady-state migration of immune cells within and between lymphoid organs, the bloodstream, and peripheral tissues as part of immune surveillance. CXCL12 also plays a key role in retaining neutrophils and other leukocytes to the bone marrow. However, when synergizing with CXCL8 or other chemokines, it attracts B- and T-lymphocytes, dendritic cells, and monocytes (62). Two studies reported significantly elevated CXCL12 levels in AA skin, while one study found it to be significantly lower in blood (2.9-fold) (18, 27, 63).

#### XCL1 and XCL2

The XCL1–XCR1 axis plays a key role in cytotoxic immunity. XCR1 is expressed on conventional type 1 dendritic cells (cDC1s), which are essential for priming CD8+ cytotoxic T cells (64, 65). It was not increased in serum, while two studies showed a significant increase in AA tissue (4.64-fold, p<0.05) (13, 27). The related chemokine XCL2 was studied in only 1 report, showing significantly increased levels in AA skin (3.8-fold, p<0.05) (13).

#### CCL28

The mucosae-associated epithelial chemokine CCL28 attracts Tregs and eosinophils (66). It was only reported by 1 study, showing slightly lower concentrations in the serum of AA patients compared to controls (-1.03-fold, no significance) (13).

**Figure 4:**
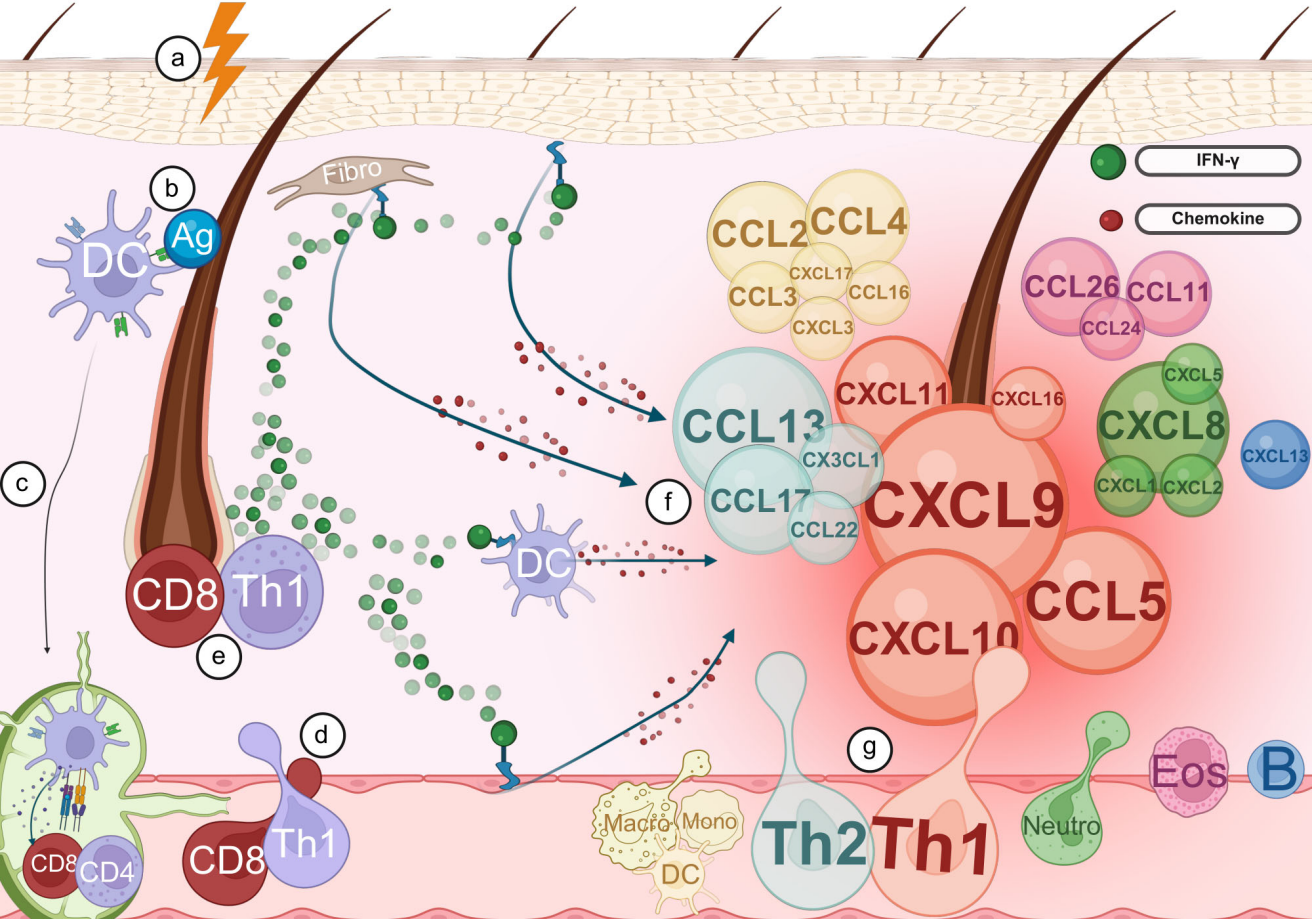
Chemokines involved in immune cell recruitment to the hair follicle in alopecia areata. **(a)** Trigger leads to the breakdown of immune privilege (IP) in the hair follicle. **(b)** The breakdown of IP in the hair follicle results in abnormal exposure of self-antigens (Ag). **(c)** Dendritic cells (DCs) capture the exposed antigens and migrate to the lymph nodes where they activate naive T cells. **(d)** Activated T cells exit the lymph nodes and travel via the bloodstream toward the hair follicle. **(e)** Upon arrival at the hair follicle, activated T cells release IFN-γ. **(f)** Keratinocytes, fibroblasts, dendritic cells, and endothelial cells detect IFN-γ and respond by secreting a range of chemokines. The size of the chemokines depicted in the figure correlates with the strength of the evidence from our review. **(g)** Chemokines promote the recruitment of additional immune cells to the hair follicle, amplifying the local immune response. Created with BioRender.com.

**Figure 4** provides a summary of how these chemokines facilitate the recruitment of immune cells to the hair follicle, thereby contributing to the pathogenesis of AA. Chemokines are grouped by immune axis using color, and their size reflects the strength of the supporting evidence identified in our review.

## Discussion

Our review identified strong Th1 signaling in AA, marked by elevated expression of CXCL9, CXCL10, CXCL11, and CCL5 both locally and systemically. This Th1 dominance is further supported by meta-analysis and aligns with the immune patterns seen in other IFN-γ-driven diseases like vitiligo (67).

In contrast to the findings in vitiligo, Th2 signaling was prominent as well, with consistent upregulation of CCL13, CCL17, CCL22, and CX3CL1. This Th2 signature aligns with the previously mentioned frequent co-occurrence of atopy and the observed positive response to dupilumab in atopic AA patients. Moreover, levels of CCL13, CCL17, and CCL22 significantly decreased following effective dupilumab treatment while strongly correlating with reductions in SALT scores. Though these findings were more pronounced in atopic AA patients with elevated IgE levels, non-atopic patients also showed some improvements in SALT scores and reduced Th2 chemokine levels following dupilumab, although almost complete hair regrowth was rarely observed in the latter group (29). Interestingly, children have a more Th2-skewed immunity compared to adults and preliminary evidence suggests that even children with low IgE levels can respond to dupilumab, although a long-term treatment seems required before efficacy is observed (+/- 1 year) (68). Such an approach could be particularly appealing given the less favorable side effect profiles of other biological treatments, such as JAK inhibitors. Unfortunately, although published chemokine profiles in adults support this working mechanism, data on chemokine expression in children with AA remain limited.

CCL20, the signature chemokine for Th17 recruitment, was found to be elevated in the serum of AA patients, yet not in the skin (22, 25). There is moderate evidence for elevated levels of chemokines that recruit dendritic cells, monocytes, neutrophils, eosinophils, and B lymphocytes in AA, although their expression appears lower than the strong Th1 and Th2 signals and they have been less extensively studied. A surprising finding is the marked elevation of the neutrophil-attracting CXCL8 in both serum and lesional skin of AA patients, even though neutrophils are not typically involved in AA pathology and there are no clinical signs of neutrophilic inflammation. One possible explanation for the elevated CXCL8 levels is secondary upregulation in response to pro-inflammatory cytokines like IL-1β, IFN-γ, and TNF-α (69). The absence of significant neutrophil infiltration, despite elevated CXCL8, may be due to the concurrent increase in CXCL12, which retains leukocytes in the bone marrow, suggesting that their opposing effects may dampen neutrophil recruitment to the skin. Notably, beyond their opposing roles in neutrophil trafficking, CXCL8 and CXCL12 together may synergistically recruit T cells and monocytes (62). Another unexpected finding is the elevated CCL18 levels in AA, despite its typical downregulation by IFN-γ. These findings underscore the complex immune profile observed in AA, reflecting a multifaceted inflammatory environment.

8 studies examined the association between serum chemokine levels and disease severity, with variable results. Serum levels of CCL13, CCL7, CCL17, CCL27, and CXCL8 were associated with disease severity, while other chemokines were not (11, 16, 17, 19, 31, 39, 43, 55). Notably, these findings were based on single time point assessments and only one study reported dynamic changes in chemokine levels during disease progression - showing that CXCL9 and CCL5 increased during exacerbations and decreased during remission (19). In the skin, only 2 studies explored the relationship between chemokine expression and SALT scores, both reporting no correlation. However, following effective treatment with dupilumab, nearly all chemokines correlated with changes in SALT (29, 31).

Given their early involvement in disease pathogenesis, chemokines have also been studied as potential therapeutic targets. In murine models, chemokine-directed interventions have yielded encouraging results, including CXCL12-neutralizing antibodies, blockade of the CX3CR1/CX3CL1 fractalkine axis, CCR5 inhibition using maraviroc, and anti-CXCR3 antibodies (8, 28, 35, 70, 71).

Despite the overall consistency of results, some methodological limitations must be acknowledged. Regarding study quality, of the 46 included articles, 5 are abstracts and 3 are letters. Most of these focus on a limited number of chemokines, and in nearly all cases, their findings are supported by multiple full-length, peer-reviewed studies. Exceptions that warrant more cautious interpretation include CXCL12 (2 out of 3 sources are non–full articles), CCL23 (1 out of 2) and CXCL1 (1 out of 3). However, since these chemokines were reported by only a few studies, they received limited attention in our manuscript and did not influence the overall conclusions. A more detailed assessment of the potential impact of the abstracts and letters is provided in the **Supplementary Material**.

In the meta-analysis, 3 out of 10 articles were letters. As presented in the **Supplementary Material**, sensitivity analysis showed no significant changes, except for CXCL10. However, this was mainly due to the study of Wang et al. (2023), which shows contrasting results compared to other studies for several chemokines (including CXCL9, CCL5, CCL2, and CCL3). This study included COVID-induced alopecia areata which might explain differences in chemokine production. When this study was not taken into account CXCL10 was also significant without reports published as letters or short reports.

Regarding data heterogeneity of the meta-analysis, all ten studies used serum-based immunoassays and compared AA patients to healthy controls, yet considerable variability was present (I² = 83–97%). We explore potential sources of this below.

In terms of methodology, Geng shows the greatest variation, focusing on a pediatric population, while the other studies examined adults. Several studies explicitly excluded patients who had received systemic or topical treatments prior to blood sampling (e.g., Bilgic, Kuwano, Geng), whereas others did not report treatment status or applied no clear exclusion criteria (e.g., Maouia, Barahmani, Elzawhary). Some studies excluded individuals with comorbid autoimmune or inflammatory conditions, while others did not specify such exclusions. One study (Wang, 2023) included only new-onset AA cases with a suspected post-COVID vaccination etiology.

In terms of laboratory methods, all studies used some form of immunoassay; however, there was technical variation. 5 studies used classical ELISA (Bilgic, Maouia, Zainodini, Waśkiel-Burnat, Elzawahry), 4 used multiplex bead-based assays (Kuwano, Wang (2023), Geng, Barahmani), and 1 (Song) used an electrochemiluminescence (ECL) immunoassay.

Although these differences contribute to a degree of clinical heterogeneity, studies showed consistent directional effects in serum chemokine levels, suggesting a robust underlying biological signal. To correct for the high level of heterogeneity across studies, we chose a random-effects model over a fixed-effects model. This approach accounts for both within- and between-study variation and provides a more reliable estimate of the overall effect. Nevertheless, future meta-analyses may benefit from stratified analyses based on treatment status, disease chronicity, or age group, should more homogeneous datasets become available.

## Conclusion

In conclusion, this review and meta-analysis clarifies the complex chemokine profile of AA. While a dominant Th1 signal is evident, Th2 and other immune pathways are also clearly involved. This multifaceted immune landscape may contribute to AA’s therapeutic resistance and highlights the potential of broad immunomodulatory approaches—including key regulators such as the Aryl Hydrocarbon Receptor (AhR) (72).

Given their elevated serum levels in AA patients compared to healthy controls, several chemokines identified in this review show potential as biomarkers for disease activity. However, longitudinal studies remain scarce. Future research should focus on tracking chemokine dynamics over time to develop predictive models that signal upcoming flares or remission—an especially valuable tool in AA, where clear clinical warning signs are lacking and disease unpredictability places a high psychological burden on patients.

Our findings further support the role of the Th2 axis in a subset of patients. IgE levels and Th2-related chemokines have been demonstrated to predict the response of alopecia areata to dupilumab (29). At our department, we already screen for elevated IgE levels in atopic AA patients to guide the use of dupilumab, and these results reinforce the rationale for such targeted approaches. Finally, chemokine profiling may serve as a powerful tool for large-scale immune stratification, potentially informing both prognosis and personalized therapy development.

## Data Availability

The original contributions presented in the study are included in the article/**Supplementary Material**. Further inquiries can be directed to the corresponding author.
