# Supplementary figures and images for: A meta-analysis of chemokines in alopecia areata: recruiting immune cells toward the hair follicle
Source: Front Immunol. 2025 Sep 3;16:1648868. doi: 10.3389/fimmu.2025.1648868 (PMC12440783; doi:10.3389/fimmu.2025.1648868)

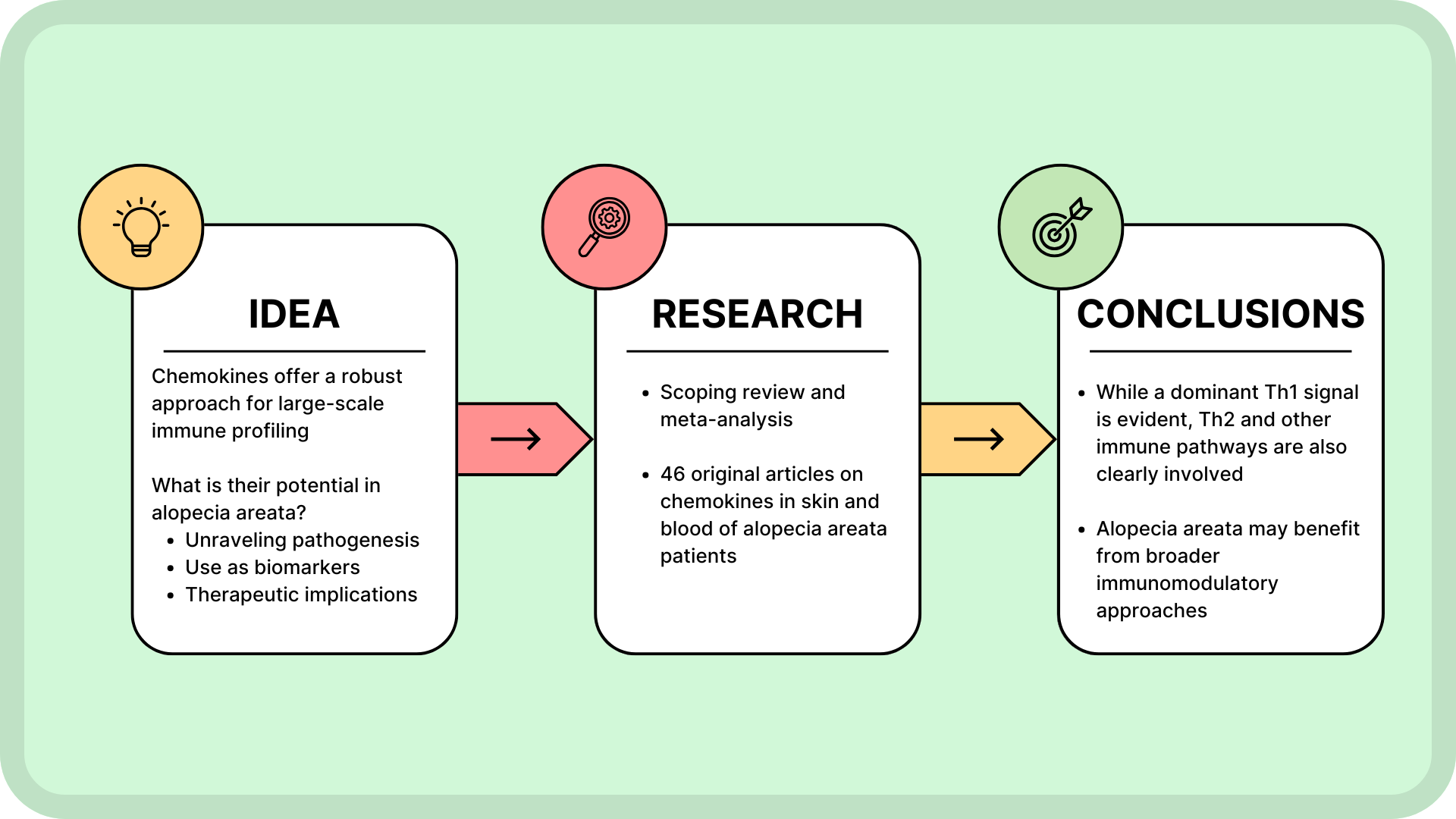

Supplement: Supplementary file 1 [file Image1.png]
