# Supplementary material for: A meta-analysis of chemokines in alopecia areata: recruiting immune cells toward the hair follicle
Source: Front Immunol. 2025 Sep 3;16:1648868. doi: 10.3389/fimmu.2025.1648868 (PMC12440783; doi:10.3389/fimmu.2025.1648868)

**Supplementary material: Assessment of study quality**

Publication types

Of the 46 articles included, only 5 are abstracts and 3 are letters. Most of these focus on a limited number of chemokines, and in nearly all cases, their findings are supported by multiple full-length, peer-reviewed studies.

One of the 5 abstracts is the study by Subramanya, which reports on CCL5, CX3CL1, CXCL10, and CXCL1. However, as it only compared lesional to non-lesional skin without including a healthy control group, it was excluded from the main comparative analysis in this review. The other 4 abstracts and 3 letters are summarized in the table below. As shown, these non–full-length publications had no meaningful impact on the conclusions of our review. Notable exceptions that require more cautious interpretation include CXCL12 (2 out of 3 sources are non–full articles), CCL23 (1 out of 2) and CXCL1 (1 out of 3). However, since these chemokines were reported by only a few studies, they received limited attention in our manuscript and did not influence the overall conclusions.

This table is best interpreted alongside Figure 2 of the manuscript.

Abstracts

| Author | Chemokines discussed | Comments |
| --- | --- | --- |
| Tabatabaei-Panah | CCL2 | Of the 8 articles on CCL2, this is the only one presented as an abstract, and its findings are consistent with those of the other studies. |
| Uchida | CXCL12 | 2 out of the 3 articles discussing CXCL12 are not full articles (this abstract and the letter from Zainodini below), so the findings on CXCL12 should be interpreted with caution. |
| Wang (2016) | CCL5, CCL4 | CCL5: Of the 12 sources on CCL5, only this abstract is not a full-length peer-reviewed article. However, the findings are consistent with those of the full publications.  CCL4: Of the 8 sources reporting on CCL4, only this abstract and the letter from Waskiel-Burnat (mentioned below) are not full-length peer-reviewed articles. While Waskiel-Burnat reports findings consistent with those of the full publications, Wang’s results differ, reporting a decrease in CCL4 levels in AA where most other studies report an increase. |
| Wang (2021) | CXCL9, CXCL10, CCL13 | CXCL9: Of the 17 sources reporting on CXCL9, only this abstract and the letter by Zainodini (mentioned below) are not full-length peer-reviewed articles. Both, however, report findings that are consistent with those of the full publications  CXCL10: Of the 17 sources reporting on CXCL10, only this abstract and the letter by Zainodini (mentioned below) are not full-length peer-reviewed articles. Both, however, report findings that are consistent with those of the full publications  CCL13: Of the 11 articles on CCL13, this is the only one presented as an abstract, and its findings are consistent with those of the other studies. |

Letters

| Author | Chemokines discussed | Comments |
| --- | --- | --- |
| Waśkiel-Burnat | CCL7, CCL4 | CCL7: Of the 5 studies investigating CCL7, this is the only letter, and its results align with the findings reported in the full articles.  CCL4: Of the 8 sources reporting on CCL4, only this letter and the abstract by Wang (listed in the table above under 'abstracts') are not full-length peer-reviewed articles. While Waskiel-Burnat reports findings consistent with those of the full publications, Wang’s results differ, reporting a decrease in CCL4 levels in AA where most other studies report an increase. |
| Wu | CCL23 | Only 2 articles report on CCL23, one of which is this letter. Therefore, the findings should be interpreted with caution. |
| Zainodini | CXCL9, CXCL10, CXCL12, CXCL1 | CXCL9: Of the 17 sources reporting on CXCL9, only this letter and the abstract by Wang (listed in the table above under 'abstracts') are not full-length peer-reviewed articles. Both, however, report findings that are consistent with those of the full publications.  CXCL10: Of the 17 sources reporting on CXCL10, only this letter and the abstract by Wang (listed in the table above under 'abstracts') are not full-length peer-reviewed articles. Both, however, report findings that are consistent with those of the full publications.  CXCL12: 2 out of the 3 articles discussing CXCL12 are not full articles (this letter and the abstract from Uchida above), so the findings on CXCL12 should be interpreted with caution.  CXCL1: Only 3 articles report on CXCL1, one of which is this letter. Therefore, the findings should be interpreted with caution. |

Sensitivity analysis

In the **meta-analysis component** of our review, 3 out of 10 articles were letters. As presented below, sensitivity analysis showed no significant changes, except for CXCL10. However, this was mainly due to the study of Wang et al. 2023 which shows contrasting results compared to other studies for several chemokines (including CXCL9, CCL5, CCL2, CCL3). This study included COVID-induced alopecia areata which might explain differences in chemokine production. When this study was not taken into account CXCL10 was also significant without reports published as letters or short reports.

CXCL9


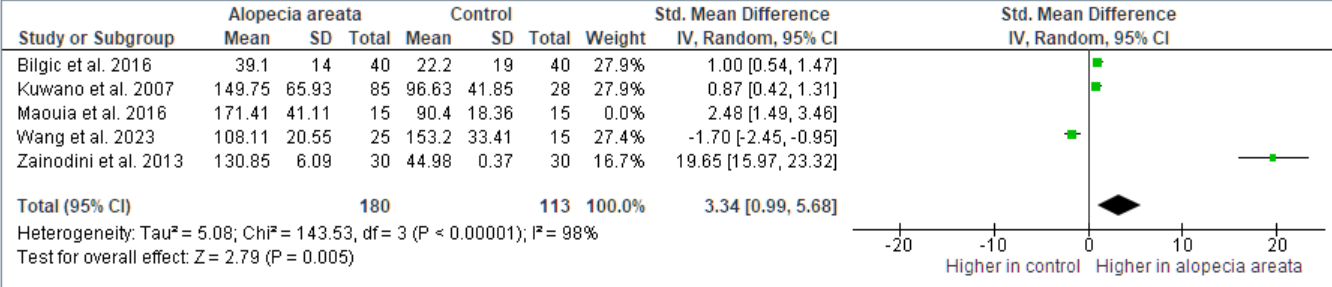


CXCL10


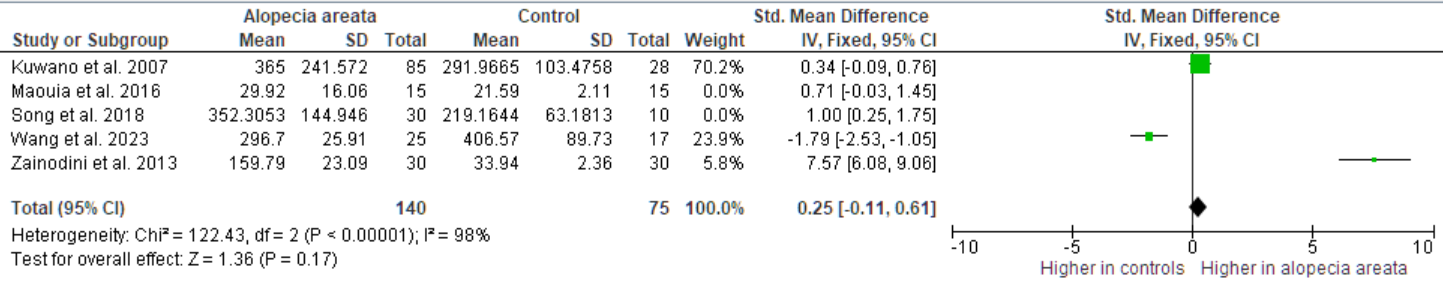


Without Wang et al 2023


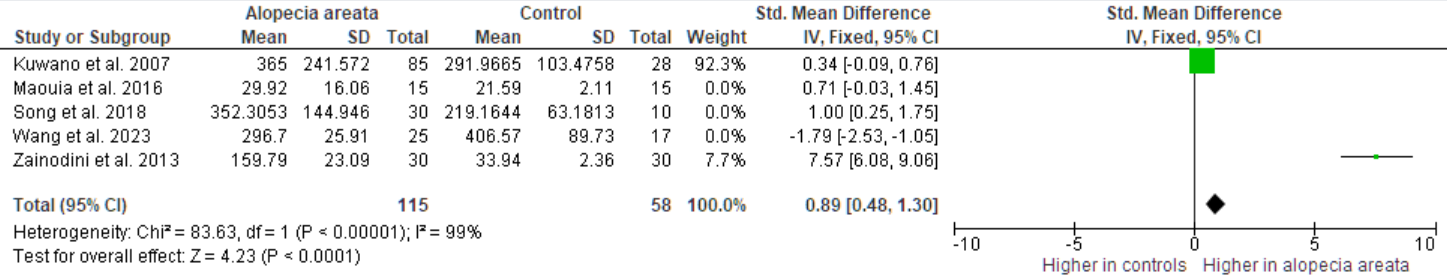


CXCL8


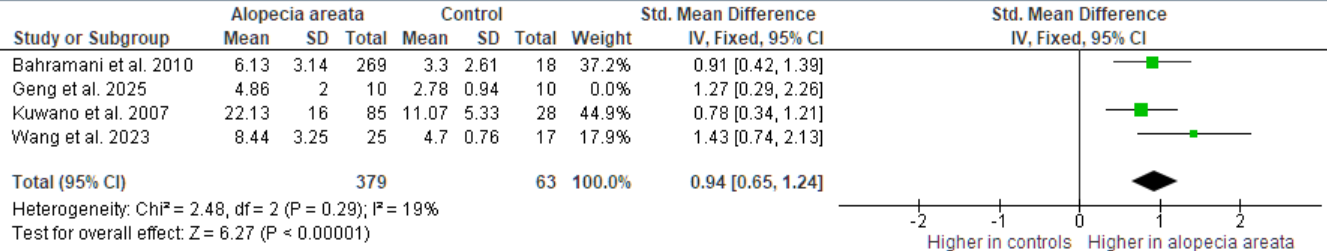


CCL17


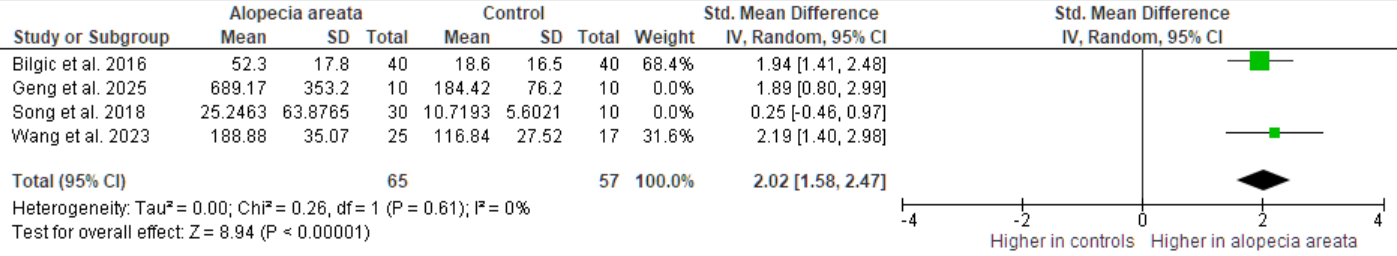


CCL5


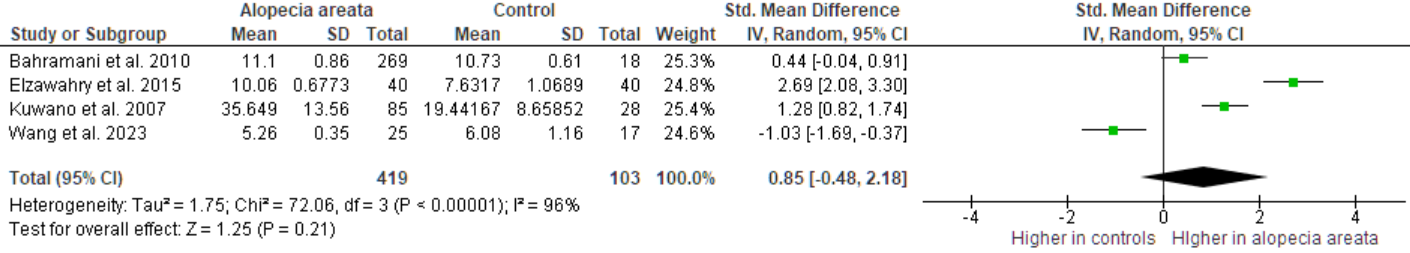


CCL2


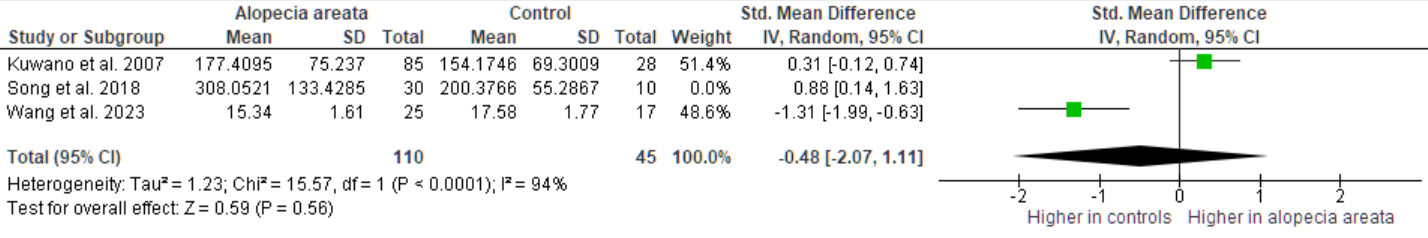


CCL3


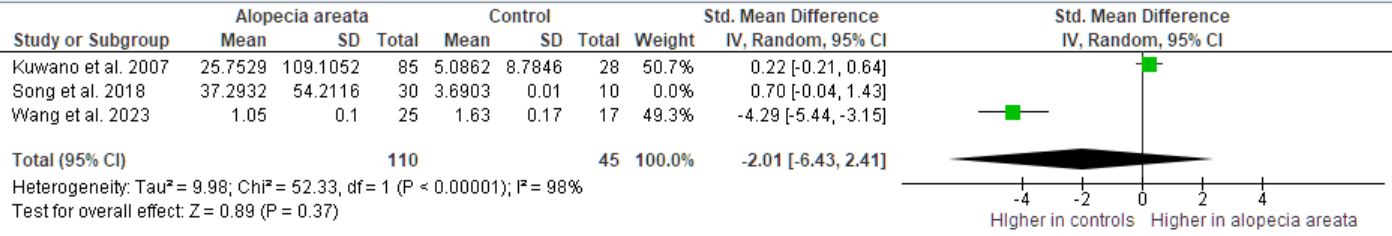


CCL4


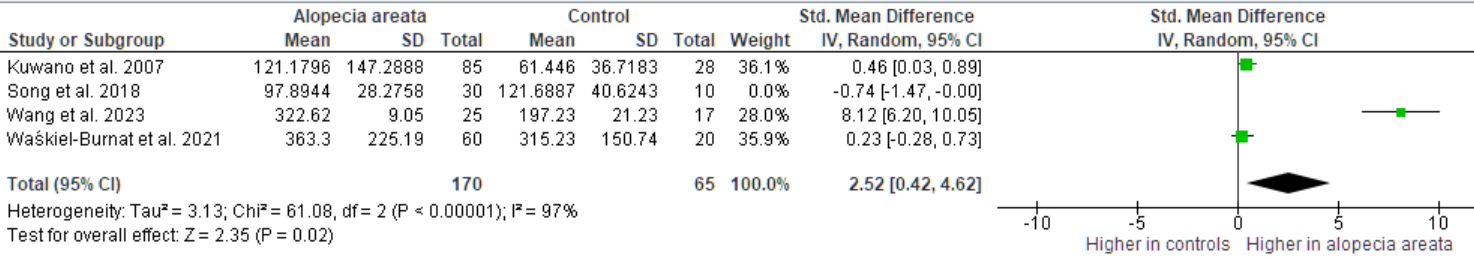

Supplement: Supplementary file 2 [file Table1.docx]
