# Supplementary material for: A meta-analysis of chemokines in alopecia areata: recruiting immune cells toward the hair follicle
Source: Front Immunol. 2025 Sep 3;16:1648868. doi: 10.3389/fimmu.2025.1648868 (PMC12440783; doi:10.3389/fimmu.2025.1648868)
Supplement: Supplementary file 3 [file Table2.docx]

**Supplementary material: Plain language summary**

Alopecia areata (AA) is a condition where the immune system mistakenly attacks hair follicles, leading to hair loss. This can affect small patches or, in more severe cases, cause complete loss of hair on the scalp or body. Although scientists know that the immune system is involved, the exact reasons why this happens are still not fully understood.

To better understand how a group of immune messengers called **chemokines** may be involved, we carried out a large review of published research. Chemokines are small proteins that help guide immune cells to specific areas of the body. Unlike other immune signals called cytokines, chemokines are found in higher levels in the blood, making them easier to study.

We looked at 46 scientific studies that measured chemokines in the blood and skin of people with AA. Some of these chemokines were consistently found in higher amounts, especially those linked to a type of immune response called Th1. While this Th1 pathway is known to be involved in AA, we also found signs of other immune activity, including Th2 responses and markers related to allergic inflammation. This suggests that AA involves a complex mix of immune signals, beyond the classical Th1 pathway.

These findings suggest that chemokines could be useful not only as markers of disease activity but also as potential treatment targets—not just for the Th1 pathway, but also for Th2 and other immune pathways involved in AA.
